# Partially hidden multi-state modelling of a prolonged disease state defined by a composite outcome

**DOI:** 10.1007/s10985-018-09460-y

**Published:** 2019-01-19

**Authors:** Vernon T. Farewell, Li Su, Christopher Jackson

**Affiliations:** MRC Biostatistics Unit, School of Clinical Medicine, University of Cambridge, Robinson Way, Cambridge, CB2 0SR UK

**Keywords:** Composite outcome, Hidden states, Minimal disease activity, Multi-state models, Psoriatic arthritis

## Abstract

For rheumatic diseases, Minimal Disease Activity (MDA) is usually defined as a composite outcome which is a function of several individual outcomes describing symptoms or quality of life. There is ever increasing interest in MDA but relatively little has been done to characterise the pattern of MDA over time. Motivated by the aim of improving the modelling of MDA in psoriatic arthritis, the use of a two-state model to estimate characteristics of the MDA process is illustrated when there is particular interest in prolonged periods of MDA. Because not all outcomes necessary to define MDA are measured at all clinic visits, a partially hidden multi-state model with latent states is used. The defining outcomes are modelled as conditionally independent given these latent states, enabling information from all visits, even those with missing data on some variables, to be used. Data from the Toronto Psoriatic Arthritis Clinic are analysed to demonstrate improvements in accuracy and precision from the inclusion of data from visits with incomplete information on MDA. An additional benefit of this model is that it can be extended to incorporate explanatory variables, which allows process characteristics to be compared between groups. In the example, the effect of explanatory variables, modelled through the use of relative risks, is also summarised in a potentially more clinically meaningful manner by comparing times in states, and probabilities of visiting states, between patient groups.

## Introduction

For studies in rheumatic diseases, and in other medical contexts, the outcome variable of interest is often composite. Such an outcome will be defined based on the observed values of a set of separate variables that all reflect some aspect of a patient’s disease activity. Sometimes the composite outcome is a mathematical function of the values of the constituent variables and sometimes it may be a categorical variable representing disease states defined in terms of the constituent variables. In this paper, we focus on the latter situation.

It may also be the case that clinical interest focuses on a patient being in a disease state for a prolonged period of time. For example, the concept of minimal disease activity (MDA) in rheumatic disease was conceptually defined as “that state of disease activity deemed a useful target of treatment by both the patient and physician, given current treatment possibilities and limitations” by The Outcome Measures in Rheumatology Clinical Trials 6 Conference (Wells et al. 2005). This reflects the fact that the complete absence of disease is not a realistic goal for many patients. For psoriatic arthritis (PsA), an inflammatory arthritis associated with the skin disease psoriasis, this has been operationally defined in terms of 7 criteria related to physician, patient and laboratory measures of disease activity, that is, disease symptoms that are potentially reversible through treatment or other factors. However, short term MDA is of little clinical interest as it is MDA of extended duration, typically one year or more, that has been linked with reducing the risk of permanent joint damage, a major aspect of disease progression in PsA (Coates et al. 2010a).

There are challenges to the analysis of events that are defined by prolonged observation of a condition (Farewell and Su 2011) and relatively simple approaches are often adopted in practice. For example, Coates et al. (2010a) divided a longitudinal cohort of patients into two groups, those who achieved the criteria for MDA at consecutive visits for a minimum of 12 months and those who did not over their periods of followup. These two groups were then compared in various ways in terms of explanatory variables. This approach does not appear to take full advantage of the longitudinal follow-up of the patients or reflect the intermittent observation patterns of the cohort. Along with the need for more comprehensive longitudinal modelling reflecting intermittent observation of patients, typically at clinic visits, a sizeable number of clinic visits may not provide information on a sufficient number of MDA criteria to unambiguously determine whether a patient is in the MDA state. In this paper, we examine how these challenges may be met when adopting a simple two-state model for the presence and absence of MDA in PsA. The primary aim is to provide a means to characterise the MDA process in PsA. An additional benefit is that this model can be extended to incorporate explanatory variables, which allows process characteristics to be compared between groups. The effect of explanatory variables, modelled through the use of relative risks, can also be summarised in a potentially more clinically meaningful manner by comparing times spent in states, and probabilities of visiting states, between patient groups.

As in Sweeting et al. (2010), features of the observation process suggest the use of a partially hidden multi-state model. Aalen (2010) commented on this previous work that the model formulation was needed to address the nature of the available data. He commented on the use of Markov formulations as “a simple way of introducing dynamics into the system” and that while “the Markov assumption is often criticized as being too strong ...  a simple Markov assumption will, for many purposes be good enough”. In addition, our incorporation of available data from visits when MDA can not be unambiguously determined, is also similar to the use of an auxiliary variable in Sweeting et al. (2010) to address the problem of informative observation, another consideration highlighted in Aalen (2010). Thus the modelling approach discussed in this paper is, we hope, taking account of the issues raised in Aalen (2010) and it is a great pleasure to contribute the paper to this special issue of the journal prepared in honour of Odd Aalen’s long and distinguished research career.

## The clinical example

**Table 1:** Numbers of visits, by number of MDA-defining criteria observed and number of these which were positive, by MDA status

Number of criteria positive	Number of criteria observed							
	0	1	2	3	4	5	6	7
*No MDA*								
0	0	0	0	79	15	19	40	13
1	0	0	0	0	119	137	210	74
2	0	0	0	0	0	294	411	151
3	0	0	0	0	0	0	619	224
4	0	0	0	0	0	0	0	266
5	0	0	0	0	0	0	0	0
6	0	0	0	0	0	0	0	0
7	0	0	0	0	0	0	0	0
*MDA*								
0	0	0	0	0	0	0	0	0
1	0	0	0	0	0	0	0	0
2	0	0	0	0	0	0	0	0
3	0	0	0	0	0	0	0	0
4	0	0	0	0	0	0	0	0
5	0	0	0	0	0	174	566	226
6	0	0	0	0	0	0	344	211
7	0	0	0	0	0	0	0	202
*Indeterminate MDA status*								
0	8	1	56	0	0	0	0	0
1	0	15	14	76	0	0	0	0
2	0	0	26	89	229	0	0	0
3	0	0	0	113	346	474	0	0
4	0	0	0	0	215	351	617	0
5	0	0	0	0	0	0	0	0
6	0	0	0	0	0	0	0	0
7	0	0	0	0	0	0	0	0

Our motivating example is based on data from 7024 clinical visits from 856 patients seen at the University of Toronto PsA Clinic since 2003. Patients were evaluated using a standard protocol every 6–12 months. Patients were followed up for a median time of 3 years (maximum 10 years), with a median 6 visits (maximum 27). This intermittent observation pattern needs to be reflected in analyses, as discussed earlier, but it is important to note that visits occurring within 3 months of a regularly scheduled visit, perhaps to address clinical needs identified at the previous visit, are not included in the database. Clinical assessments included the number of (out of 68) tender joints and the count of (out of 66 excluding hips) swollen joints, a measure of enthesitis reflecting the number of inflamed locations where tendons or ligaments insert into bones, and a dactylitis score reflecting the extent to which entire digits are inflamed, a characteristic symptom of PsA. Skin assessment included both the body surface area (BSA) and the Psoriasis Area and Severity Index (PASI) which has a range 0–72. A clinically count of permanently damaged joints was also recorded at each visit. A physician global assessment on a 10 cm scale was to be completed at every visit and patients completed self-reported questionnaires including the Health Assessment Questionnaire (HAQ), which has a range of 0–3, and patient global assessments, on a 10 cm scale, usually at every other visit.

The criteria for the definition of MDA used by Coates et al. (2010b) were 5 or more out of 7 of the following:

1. Tender joint count (TJC) $\le$1
2. Swollen joint counts (SJC) $\le$ 1
3. PASI score $\le$1 or BSA $\le$ 3%
4. Patient pain visual analog score (PTPPAINV) $\le$ 1.5 cm
5. Patient global disease activity visual analogue score (PTPSA) $\le$ 2 cm
6. HAQ score $$\le $$ 0.5
7. Entheseal points (ENTH_TOT) $\le$1

All but 8 clinic visits provided information on at least one of the MDA criteria, and the numbers with 0 to 7 criteria missing were 1367, 2807, 1449, 924, 357, 96, 16 and 8 respectively. The HAQ and patient global scores were missing approximately 50% of the time as per their scheduled administration at every other visit and the rest were missing at 10% to 20% of visits. The MDA status could be determined at 63% of the visits comprising 38% when at least 5 out of 7 criteria were observed and satisfied and 25% when at least 3 out of 7 were observed and not satisfied. There were 1390 and 195 occasions when MDA was observed followed by no MDA and MDA, respectively, at the next visit, and there were 144, and 825 occasions when no MDA was observed, followed by no MDA, and MDA, respectively, at the next visit. Table 1 presents the number of positive criteria observed by MDA status and the number of criteria observed.

For the purposes of regression analyses, in which the effect of baseline explanatory variables on MDA is subsequently examined, if no treatment information is recorded for a patient then it has been assumed that neither disease modifying anti-rheumatic drugs (DMARDs) or biologic agents were given. Patients with missing information on any baseline explanatory variable were excluded. The highest fraction of missingness, 9%, was seen with the binary indicator for the involvement of axial joints. The other indicator variables used in our example analyses were for polyarthritis (the involvement of five or more joints), sex, an elevated sedimentation rate (ESR) and previous damaged joints. Age and disease duration prior to clinic entry were also included as continuous variables.

## A partially hidden multi-state model

### The model

**Fig. 1:**
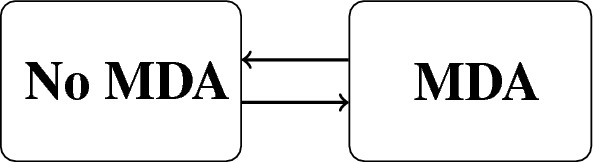
Simple two-state MDA model

Figure 1 presents the simple two-state model with states ‘MDA’ and ‘No MDA’. The model is characterised by two transition rates, one for the transition from ‘No MDA’ to ‘MDA’ and the other for ‘MDA’ to ‘No MDA’. Specifically, we will fit a time-homogeneous Markov multi-state model with constant transition intensities for the MDA process.

For each patient, let $S_{j}$ represent their MDA status at clinic visit *j*, and let $y_{jk}$ represent the observed value of the *k*th variable used to define MDA status at clinic visit *j*. Because two variables may be used to determine the third MDA criterion described in Sect. 2, there are 8 defining variables in total. Additionally, let $\mathbf {x}_{j}$ represent a row vector of explanatory variables associated with the patient at visit *j*. The transition rates can then be specified as

1 $$\begin{aligned} \lambda _ {({\text {No}\rightarrow \text {MDA}})}(\mathbf {x}_{j}) = \lambda _ {({\text {No}\rightarrow \text {MDA}}, 0)} \exp (\mathbf {x}_{j}{\beta }) \end{aligned}$$

and

2 $$\begin{aligned} \lambda _ {({\text {MDA}\rightarrow {\text {No}}})}(\mathbf {x}_{j}) = \lambda _ {({\text {MDA}\rightarrow {\text {No}}}, 0)} \exp (\mathbf {x}_{j}{\gamma }), \end{aligned}$$

where $\lambda _ {({\text {No}\rightarrow \text {MDA}}, 0)}$ and $\lambda _ {({\text {MDA}\rightarrow {\text {No}}}, 0)}$ are baseline intensities, ${\beta }$ and ${\gamma }$ are column vectors of regression coefficients associated with the explanatory variables in the two models. Note that the model is specified in continuous time although observation is intermittent at clinic visits.

Equations (1) and (2) reflect the necessary simplifying assumptions for a time-homogeneous Markov multi-state model with constant transition intensities, although this may be relaxed. The time-homogeneity assumption can be relaxed easily with available software through the use of piece-wise constant transition intensities. This might be needed, for example, if the introduction of new treatments resulted in variation of MDA frequency with calendar time. Departures from the Markov assumption would introduce more complications since state entry times are unknown, though some non-Markov models can be fitted to data of this kind in available software using phase-type sojourn distributions (Titman and Sharples 2010). This is expanded upon in Sect. 5. The Markov assumption is particularly useful for fitting a partially hidden multi-state model and for calculating summary characteristics of the MDA process. It is not expected that the Markov assumption which introduces the dependence of the future on the past through conditioning on current state would be an undue simplification in the context of PsA. With respect to the use of this model, in comparison to the very simple models used previously (Coates et al. 2010b), we would regard the multi-state model structure to be a critical assumption to adequately model the MDA process, but the specific Markov assumption to be a simplifying assumption regarding a secondary aspect of the model, following the approach to assumptions outlined by Cox and Snell (1981).

Because MDA status can not be determined at all visits, it is convenient to regard this model as a partially hidden multi-state model. At some visits the MDA status is known but at others it is unknown or hidden. This essentially extends the usual multi-state modelling approach to allow information from the $y_{jk}$ variables to provide extra information on MDA status at visits when the binary classification of MDA based on the $y_{jk}$ variables cannot be unambiguously determined. This could be done by incorporating modelling of the conditional distributions of the binary criteria derived from the $y_{jk}$ variables, given MDA status, but we will focus only on the somewhat more general approach of directly modelling the conditional distributions of the 8 $y_{jk}$ variables that determine these binary criteria. A comparison of these two approaches can be found in Jackson et al. (2016) where there is some evidence that modelling the $y_{jk}$ variables can provide greater precision for estimation of parameters in the multi-state model, as might be expected.

It is assumed that given the (observed or latent) MDA status, the distributions of the $y_{jk}$ variables are independent from each other. In other words, we assume that the marginal distributions of $y_{jk}$ variables help to discriminate the MDA status, but the associations between $y_{jk}$ variables will not provide additional information for this discrimination. Without this conditioning, an independence assumption would be unreasonable but it is less problematic given the conditioning, although it is unlikely to be exactly true.

In terms of missing data for $y_{jk}$, we assume that the unobserved $y_{jk}$ variables are missing at random given the observed $y_{jk'}$ ($k \ne k'$) values at visit *j*. Therefore, we don’t model missing indicators of $y_{jk}$ and relate them to the latent MDA status. If missingness depends on the unobserved $y_{jk}$ values after conditioning on the observed data, then the missing indicators should also inform the latent MDA status and need to be modelled. This will correspond to a *latent ignorability* assumption discussed in Harel and Schafer (2009). Because a substantial amount of partially missing data in the PsA clinic are due to different schedules for data collection, e.g., HAQ is only measured every other visit, we reckon that the missing at random assumption is reasonably plausible in this context.

To specify the probabilities $\text{ Pr }(y_{jk}|S_j=r)$, the patient pain and global activity scores are rounded to integers and assumed to arise from $Binomial(10, p_{kr})$ distributions while the remaining variables, which are all integers if HAQ and PASI are multiplied by 100, are specified to arise from negative binomial distributions, $NegBin(n_k, p_{kr})$. Heuristically, information on the set of all $n_{k}$ and $p_{kr}$ parameters will arise primarily from visits when $S_{j}$ is observed, while for latent or hidden values of $S_{j}$, the subset of $y_{jk}$ values observed will provide information on the possible MDA status. Low values of $y_{jk}$ variables are more likely to be associated with an underlying MDA state.

### Estimation

The proposed model, with its Markov assumption, can be fitted by full maximum likelihood. Introducing an additional subscript *i* for patients, let $\mathbf {y}_{ij}$ represent the vector of MDA defining variables observed at visit *j* from patient *i*, where $j=1,\ldots ,n_i$ and $i=1,\ldots ,m$.

Let $\text{ Pr }(S_{ij} \mid S_{i,j-1}, \mathbf {q}, \mathbf {x}_{ij})$ be the transition probability from state $S_{i,j-1}$ to state $S_{ij}$ over the time interval separating visits $j-1$ and *j*, given explanatory variables $\mathbf {x}_{ij}$, where $\mathbf {q}= (\lambda _ {({\text {No}\rightarrow \text {MDA}}, 0)}, \lambda _ {({\text {MDA}\rightarrow {\text {No}}}, 0)},{\beta },{\gamma })$ represents both the transition rates governing the hidden Markov chain and the effects of explanatory variables on these. Let $\text{ Pr }(S_{i1} \mid \mathbf {f})$ be the distribution of (potentially unknown) MDA states at the initial visit, with vector of probabilities $\mathbf {f}$. Finally, let $f(\mathbf {y}_{ij} \mid S_{ij}, {\alpha })$ be the conditional distribution of $\mathbf {y}_{ij}$ given the states $S_{ij}=0$ (“no MDA”) and $S_{ij}=1$ (“MDA”), with the parameter vector ${\alpha }$. Then, assuming that the $\mathbf {y}_{ij}$ are conditionally independent given $S_{ij}$, the full likelihood can be represented as

3 $$\begin{aligned} l({\theta } \mid {\{S\}}, \mathbf {y}, \mathbf {x})= & {} \prod _i \sum _{\{S_i\}} \{\text{ Pr }(S_{i1} \mid \mathbf {f}) f(\mathbf {y}_{i1} \mid S_{i1}, {\alpha })\nonumber \\&\times \prod _{j>1}\text{ Pr }(S_{ij} \mid S_{i,j-1}, \mathbf {q}, \mathbf {x}_{ij}) f(\mathbf {y}_{ij} \mid S_{ij}, {\alpha })\}, \end{aligned}$$

where the parameters are ${\theta } = (\mathbf {q},\mathbf {f}, {\alpha })$, $\{S\}$ is the set of all observed MDA states, and the product over visits *j* is summed over all possible latent state pathways $\{S_i\}$ for each patient *i* (Satten and Longini 1996).

Note that the “data” in this model implicitly includes the observations of MDA status $$S_{ij}$$ at times *j* when this is known, which constrains the set of latent state pathways to be summed over. Satten and Longini (1996) showed further that the likelihood contribution from a patient *i* in this model can be expressed as a product of $$n_i$$$$K\times K$$ matrices, where *K* is the number of states in the Markov model structure ($$K=2$$ in our example), which facilitates computation. Our model generalises the model in Satten and Longini (1996) to composite outcomes given the hidden state, and a combination of observed and hidden states $$S_{ij}$$.

Maximum likelihood estimation is implemented in the msm R package (Jackson 2011; R Development Core Team 2010) for continuous-time Markov and hidden Markov modelling. The package allows general state-transition structures with transition intensities depending on explanatory variables. There can be any number of outcomes linked to a hidden state, with a variety of distributional assumptions possible. The implementation is based on derivatives of the log-likelihood (Lystig and Hughes 2002) and uses the R optim BFGS method.

### Complete case analysis

If the possible information from $\mathbf {y}_{ij}$ values observed at visits when MDA status can not be determined is ignored, then the two-state model in Fig. 1 can simply be fitted using the standard likelihood for a continuous-time Markov model for panel data (Kalbfleisch and Lawless 1985),

$$\begin{aligned} l(\mathbf {q}| {\{S\}},\mathbf {x}_{ij}) = \prod _{i,j} \text{ Pr }(S_{ij} | S_{i,j-1}, \mathbf {q}, \mathbf {x}_{ij}), \end{aligned}$$

where $\text{ Pr }(S_{ij} | S_{i,j-1},\mathbf {q}, \mathbf {x}_{ij})$ is the transition probability from observed state $S_{i,j-1}$ to state $S_{ij}$ over the time interval separating visits $j-1$ and *j* given $\mathbf {x}_{ij}$, and the likelihood contribution for each person is conditioned on their initial observation $S_{i1}$. This can again be implemented in the msm package. Note that in this model, the observations include only the 63% of patient visits at which MDA can be determined. This analysis will be termed a complete case analysis. The comparison of this with the analysis based on the partially hidden multi-state model analysis will provide some indication of whether the latter can provide any gains in precision or any bias reduction relative to the former.

### Analyses related to sustained MDA

As well as parameter estimation of a multi-state model, there is often interest in summary measures related to state occupancy. Estimation of quantities such as the expected duration of time in a state and total time in a state or the number of times in a state over a fixed time period can be derived as analytic functions of transition rates from continuous-time Markov chain theory. However, there is clinical interest in prolonged durations of state occupancy for MDA, such as the one year duration to define sustained MDA. For the estimation of these, simple analytic calculations of relevant measures are not possible.

Therefore, in order to provide information on sustained MDA, estimated expectations can be calculated by simulation of state histories over a period of time, say 10 years, for 100,000 patients under the fitted multi-state model. Specifically, the history of a patient with explanatory variable vector $\mathbf {x}_{j}$ is simulated as a series of periods alternately spent in No MDA and MDA, starting with No MDA, and each with duration simulated from an exponential distribution with rate $\lambda _{\text {No}\rightarrow \text {MDA}}(\mathbf {x}_j)$ or $\lambda _{\text {MDA}\rightarrow \text {No}}(\mathbf {x}_j)$ respectively, until a censoring point of 10 years. Then the probability of visiting sustained MDA, for example, can be estimated as the proportion of simulated patients with sojourns of one year or more in the MDA state starting before 10 years. If the patient is in MDA at 10 years, then they are followed up further until the end of the MDA sojourn, so that this sojourn can be categorised as sustained or not. Furthermore, as done in Aalen et al. (1997) for simpler functions of parameters from a multi-state model, confidence intervals and standard errors for these estimates can be determined by simulating from the distribution of parameter values, say 1000 times, and repeating the simulation of state histories for 100,000 patients for each of these 1000 sets of parameter values.

**Fig. 2:**
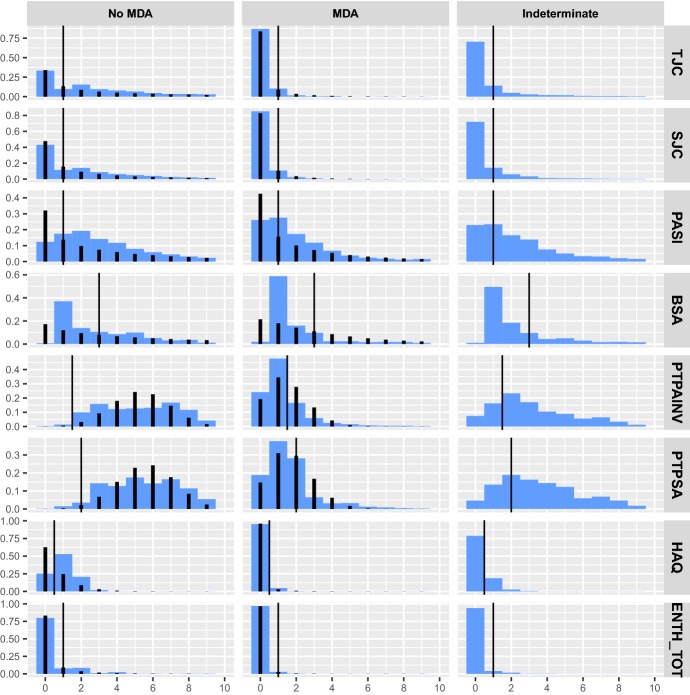
Histograms of MDA defining variables with binary cutpoints and corresponding fitted probabilities (vertical bars) from a simple two-state model: TJC: Tender joint count, SJC: Swollen joint count, PASI: Psoriasis Area and Severity Index, PTPAINV: Patient pain visual analog score, PTPSA: Patient global disease activity visual analog score, HAQ: Health Activity Questionnaire, ENTH_TOT: Inflamed entheseal points

## Results for clinical example

### Simple two-state model

Figure 2 (first and second columns) shows the observed and fitted distributions of each MDA-defining criterion conditional on known MDA states along with an indication of the binary cutpoints used to define the binary MDA defining criteria. Not surprisingly, some variables appear to have more potential to discriminate between the two states than others. Also shown in Fig. 2 are vertical bars within each histogram segment which display the maximum likelihood estimation results for these distributions. The negative binomial model can be seen to fit well for TJC, SJC and ENTH_TOT. However for PASI, BSA and HAQ, the shape of the negative binomial does not perfectly represent the spike at zero and the distribution of the non-zero values. Similarly, for PTPAINV and PTPSA, the variance of the observed distributions can be seen to differ slightly from the variance of the fitted binomial. Divergence from the histograms which could result from an inappropriate distributional assumption and/or the fact that the fitted distributions make use of additional data, as shown in columns three of Fig. 2, from patients visits at which MDA can not be determined unequivocally but which generate weighted contributions to the fit of the conditional distributions of interest. To consider the possibility of inappropriate distributional assumptions, an alternative model was explored. For PTPAINV and PTPSA, beta-binomial conditional distributions were fitted, to account for the over/under-dispersion. For the remaining three criteria, to robustify against model misspecification, these were coarsened into two binary criteria, according to the definition of MDA from Coates et al. (2010b), that is, HAQ > 0.5, and a single outcome of PASI $\le$ 1 or BSA $\le$ 3%, and binary conditional distributions were fitted. The estimate of the expected total years in MDA over 10 years under this model is 4.43, compared to 4.47, as reported subsequently, under the original model. Therefore this key result appears to be robust to specification of the conditional distributions of the outcomes.

**Table 2:** Estimates and standard errors from two-state model with no explanatory variables

	Complete cases	Data from all visits
*Mean years in one period of*		
No MDA ($1/{\hat{\lambda }}_{(\text {No}\rightarrow \text {MDA})}$)	4.06 (0.23)	2.82 (0.14)
MDA ($1/{\hat{\lambda }}_{(\text {MDA}\rightarrow \text {No})}$)	4.18 (0.29)	3.10 (0.17)
*Over 10 years (given no MDA at start)*		
Expected total years in MDA	4.05 (0.14)	4.47 (0.12)
$\ldots$ episodes lasting $\ge$ 1 year	3.90 (0.15)	4.22 (0.13)
Expected number of MDA periods	1.47 (0.06)	1.96 (0.07)
$\ldots$ lasting $\ge$ 1 year	1.16 (0.04)	1.42 (0.04)
P(visit MDA at least once)	0.92 (0.01)	0.97 (0.005)
$\ldots$ spell lasting $\ge$ 1 year	0.85 (0.01)	0.91 (0.01)

The first two lines of Table 2 provide estimated mean lengths of one period in the MDA and No MDA states as well as the associated standard errors based simply on the two estimated transition rates from the model without explanatory variables in Fig. 1. Results are provided from both the complete case analysis and the fitted partially hidden multi-state model based on data from all patient visits. It can be seen that there is an increased precision of estimation from the latter but also that the estimated times are substantially less. Thus, there is evidence of potentially notable bias in the complete case analysis. This may arise due to the variation seen between visits in the outcome variables which suggests greater movement between states than would be evident from the complete case analysis with its longer periods between observations.

The lower section of Table 2 presents estimation results for the expected total time in MDA, the expected number of MDA periods and the probability of visiting MDA at least once over a 10 year period. As well as providing results from the complete case and the partially hidden multi-state model analyses, estimates are also provided, through simulation as outlined in Sect. 3.4, for only MDA periods which last longer than one year. As expected given the results for the length of times in the states, the more complete use of the available data generates increased estimates for the expected total time in the MDA state, the expected number of periods of MDA and the probability of at least one period of MDA. And, again, as would be expected, these values are all reduced when focus is only on MDA periods of sustained length.

Note that the results in Table 2 are influenced by the 10 year horizon. For example, in the right column, the expected years in MDA of 4.47 is not the product of the average duration in MDA, 3.10, and the expected number of entries, 1.96, because of the 10 year cut-off when some patients would be expected to be in the MDA state.

### Explanatory variables

**Fig. 3:**
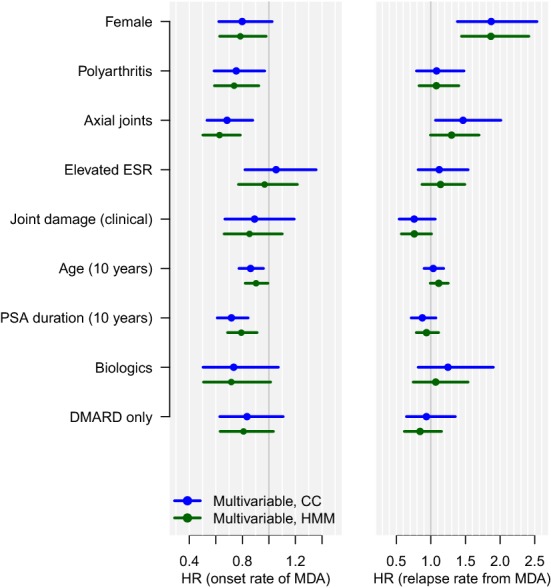
Hazard ratios, with 95% confidence intervals, from complete case analysis (CC) and partially hidden multi-state models (HMM) with multiple explanatory variables

Figure 3 presents estimated transition intensity ratios (HRs), and their 95% confidence intervals, from the multi-state model when baseline explanatory variables, as determined when first entering the cohort, are incorporated into the transition rate functions as specified in Sect. 3.1. The presentation is restricted to the results from two multivariable analyses, using complete cases and using the partially hidden multi-state model, incorporating the variables shown in Fig. 3. It can be seen that the most notable effects are associated with sex and with disease presentation as reflected in polyarthritis and axial joint involvement.

While the parameters represented in Fig. 3 derive from a very convenient relative risk model for the effects of explanatory variables, it is perhaps difficult to communicate the overall clinical implications of these effects. For example, females appear to be less likely to enter MDA and more likely to leave but it is useful to have some indication of how these effects combine to create different patterns of disease.

Tables 3 and 4 gives an illustration of how this might be done. For simplicity two single factor partially hidden multi-state model regression analyses, one including a binary indicator of female sex and the other the two binary indicators for polyarthritis and axial joint involvement are examined. These analyses, which do not adjust for other explanatory variables and therefore not conditioned on them, are not directly comparable to that presented in Fig. 3 but comparable calculations could be done for any single factor holding other factors constant using this larger model. Measures of various aspects of state occupancy for these two models are presented in Table 3 and relative measures are presented in Table 4. Some of these measures can be calculated analytically but also given are confidence intervals, all of which are derived from the simulation approach of Sect. 3.4. The more positive prognosis for males in regard to MDA can be clearly seen in the relative measures of Table 4, except for spells in MDA where the number of spells is similar for males and females, being 1.84 for males and 1.86 for females.

For the model including disease pattern variables, the relative size of the two regression coefficients seen in Fig. 3 is reflected in Table 4 by more dramatic effects for axial involvement, or both axial and polyarthritis, relative to neither disease pattern being present. Note that for both models, the effects on the odds ratio scale appear dramatic. However, this derives partially from the high probabilities of at least one MDA spell, for example 0.98 for males and 0.94 for females, so that odds ratios in this probability range can be extreme although the absolute difference in probabilities is small.

**Table 3:** MDA prognosis over 10 years between various subgroups, under two single factor partially hidden multi-state models. Model (a) includes a binary indicator of female sex only; Model (b) include two binary indicators for polyarthritis and axial joint involvement

Model (a)	Male	Female		
Sojourn time in no MDA	2.63 (2.32, 2.99)	3.55 (3.02, 4.16)		
Sojourn time in MDA	4.18 (3.59, 4.86)	2.30 (1.93, 2.75)		
Expected time in MDA	5.14 (4.81, 5.47)	3.39 (3.03, 3.76)		
Expected time in sustained MDA	3.57 (3.26, 3.87)	1.94 (1.65, 2.24)		
Time in short MDA	0.18 (0.15, 0.23)	0.30 (0.23, 0.37)		
Time in long MDA	4.95 (4.64, 5.29)	3.09 (2.75, 3.48)		
Time in first year of MDA	1.58 (1.46, 1.71)	1.45 (1.28, 1.61)		
Time in later years of MDA	3.56 (3.27, 3.86)	1.94 (1.67, 2.24)		
Spells in no MDA	2.23 (2.07, 2.41)	2.47 (2.23, 2.72)		
Spells in MDA	1.84 (1.68, 2.03)	1.86 (1.61, 2.12)		
Spells in short MDA	0.39 (0.32, 0.49)	0.66 (0.50, 0.84)		
Spells in long MDA	1.45 (1.35, 1.54)	1.20 (1.09, 1.32)		
Prob visit MDA	0.98 (0.96, 0.99)	0.94 (0.91, 0.96)		
Prob visit long MDA	0.94 (0.92, 0.96)	0.82 (0.78, 0.86)		

Model (b)	No poly or axial	Polyarthritis	Axial	Poly and axial
Sojourn time in no MDA	2.16 (1.85, 2.52)	2.81 (2.32, 3.41)	3.40 (2.80, 4.13)	4.42 (3.62, 5.40)
Sojourn time in MDA	3.83 (3.18, 4.61)	3.48 (2.82, 4.30)	2.83 (2.27, 3.54)	2.58 (1.99, 3.33)
Expected time in MDA	5.51 (5.10, 5.90)	4.67 (4.19, 5.16)	3.85 (3.37, 4.33)	3.08 (2.62, 3.56)
Expected time in sustained MDA	3.75 (3.36, 4.13)	3.09 (2.67, 3.51)	2.38 (1.98, 2.79)	1.84 (1.46, 2.25)
Time in short MDA	0.22 (0.17, 0.29)	0.22 (0.17, 0.29)	0.25 (0.19, 0.33)	0.23 (0.16, 0.32)
Time in long MDA	5.28 (4.87, 5.66)	4.47 (3.93, 4.90)	3.61 (3.10, 4.12)	2.85 (2.40, 3.37)
Time in first year of MDA	1.76 (1.60, 1.93)	1.58 (1.41, 1.78)	1.46 (1.29, 1.66)	1.24 (1.08, 1.42)
Time in later years of MDA	3.74 (3.37, 4.09)	3.11 (2.65, 3.48)	2.39 (1.98, 2.79)	1.84 (1.46, 2.29)
Spells in no MDA	2.44 (2.20, 2.72)	2.34 (2.11, 2.64)	2.36 (2.11, 2.67)	2.20 (1.96, 2.48)
Spells in MDA	2.08 (1.85, 2.34)	1.89 (1.66, 2.20)	1.81 (1.57, 2.11)	1.56 (1.33, 1.85)
Spells in short MDA	0.48 (0.37, 0.63)	0.47 (0.36, 0.64)	0.54 (0.41, 0.73)	0.50 (0.36, 0.71)
Spells in long MDA	1.60 (1.48, 1.72)	1.42 (1.29, 1.57)	1.27 (1.13, 1.42)	1.06 (0.93, 1.20)
Prob visit MDA	0.99 (0.98, 1.0)	0.97 (0.95, 0.99)	0.95 (0.91, 0.97)	0.90 (0.84, 0.94)
Prob visit long MDA	0.97 (0.95, 0.98)	0.92 (0.88, 0.95)	0.86 (0.81, 0.91)	0.77 (0.71, 0.83)

**Table 4:** Relative MDA prognosis over 10 years between various subgroups, under two single factor partially hidden multi-state models. Model (a) includes a binary indicator of female sex only; Model (b) include two binary indicators for polyarthritis and axial joint involvement

	Model (a) (relative to male)	Model (b) (relative to neither)		
	Female	Polyarthritis	Axial	Poly and axial
*Relative time*				
Sojourn time in MDA	0.55 (0.44, 0.67)	0.91 (0.75, 1.19)	0.74 (0.58, 0.99)	0.67 (0.48, 0.97)
Time in MDA	0.66 (0.58, 0.74)	0.85 (0.75, 0.94)	0.70 (0.62, 0.79)	0.56 (0.47, 0.67)
Time in sustained MDA	0.62 (0.54, 0.71)	0.84 (0.74, 0.94)	0.68 (0.59, 0.78)	0.54 (0.44, 0.66)
*Relative number*				
Spells in MDA	1.01 (0.86, 1.20)	0.91 (0.78, 1.06)	0.87 (0.73, 1.02)	0.75 (0.60, 0.93)
Spells in long MDA	0.83 (0.74, 0.93)	0.89 (0.79, 0.99)	0.79 (0.70, 0.89)	0.66 (0.56, 0.78)
*Odds ratio*				
Prob visit MDA	0.37 (0.18, 0.73)	0.35 (0.14, 0.74)	0.18 (0.07, 0.40)	0.086 (0.03, 0.23)
Prob visit sustained MDA	0.28 (0.18, 0.44)	0.43 (0.23, 0.74)	0.22 (0.13, 0.38)	0.12 (0.06, 0.23)

## Discussion

A partially hidden multi-state model provides a framework for studying intermittently observed composite outcomes such as MDA. Notably, it provides a natural way to incorporate observations from the constituent variables that define a composite outcome from observation times when not all these variables are observed and the composite outcome can not be determined. The analyses presented in this paper for the specific case of MDA in PsA illustrate the potential for this to increase precision and to protect against bias.

Coates et al. (2010a) previously examined MDA in psoriatic arthritis but as well as requiring 5 of the 7 criteria to be fulfilled, also required that MDA must be observed at consecutive visits for a minimum of 12 months in order to focus on sustained MDA. In our example dataset, which updates that of Coates et al. (2010a), and based on complete case data, 229/619 (37%) of patients achieved this and the median duration of such episodes was 42 months (3.5 years), greater than the median of 28 months presented in Coates et al. (2010a) based on earlier data on 344 patients. While there may be other reasons for this difference, the difference is at least partially explained simply on the basis of followup times as the length of MDA episodes will be censored at the last observation time. For these episodes in our data which begin prior to 2008, which is the cutoff for the data of Coates et al. (2010a), the mean duration is 76 months (6.3 years), reflecting the additional followup of the patients considered in Coates et al. (2010a). For MDA episodes in our data beginning after 2007 the mean duration is 27 months (2.3 years). Thus, estimation of the length of MDA episodes in this manner is problematic and the estimated mean durations arising from a two-state model should be preferred as these are valid estimates not influenced by followup times.

**Fig. 4:**
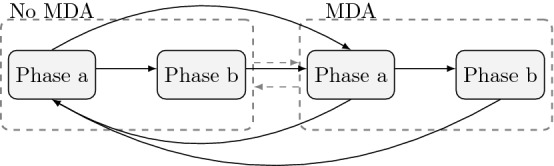
Two-phase semi-Markov model, with two states (no MDA, MDA) each with two phases. This is a hidden Markov model on the four phases, with allowed transitions indicated by solid lines. In any phase, an individual can either move to the next phase within that state, or exit to phase (a) of the next state in the Markov model it is based on (indicated by dashed lines)

As a check of the Markov assumption used in the models reported, a semi-Markov model was fitted to the data with fully-observed MDA statuses, using “phase-type” distributions. The two states are divided into two latent “phases”, resulting in a four-state hidden Markov model in Fig. 4, with 6 instead of 2 transition rates to be estimated. Thus the exponentially-distributed sojourn in each state is replaced by a sequence of either one or two sojourns with different transition rates. This allows the transition intensity from each state to change with the length of time spent in that state. The maximised likelihood changes from $-1583$ under the Markov model to $-148$1 under the semi-Markov model, while the estimated time spent in MDA over 10 years increases from 4.05 to 4.10. Given the estimates from this model, there is some evidence that the transition intensities, both to and from MDA, decrease with time spent in the current state. However a similar phase-type model with the partially-observed data would be challenging to define and identify from the data, and the principal results of interest appear to be robust to departures from the Markov assumption.

The use of a multi-state model also allows a natural way to investigate the relationship between a composite outcome and explanatory variables. The model can be parameterised in terms of relative transition intensity functions for the multi-state model. However, it is also possible to make inference on relative measures of state occupancy which may provide a more useful presentation of the effects of explanatory variables. This approach to summarising findings from the use of a multi-state model may warrant consideration in other contexts.

As in Coates et al. (2010a) where the relationship between sustained MDA and the subsequent development of permanent damage in PsA was of interest, a composite outcome measure may also be of interest in terms of its longitudinal relationship to other outcomes. It is likely to be useful in this case to make use of a partially hidden multi-state model for the composite outcome, with its more comprehensive modelling, to understand better this relationship, particularly if prediction is not the only or primary focus of the investigation. In some cases, an approach to this might be to incorporate the partially hidden multi-state model framework into a larger multi-state model with state definitions also incorporating the additional outcomes to be related to the composite outcome. This has been done for the combination of simpler multi-state models in Tom and Farewell (2011). However, this will not always practical, or the most useful, approach, so further investigation of this problem is warranted.

## References

[CR1] Aalen OO (2010). Understanding disease processes. Stat Med.

[CR2] Aalen OO, Farewell VT, De Angelis D, Day NE, Gill N (1997). A Markov model for HIV disease progression including the effect of HIV diagnosis and treatment: application to AIDS prediction in England and Wales. Stat Med.

[CR3] Coates LC, Cook R, Lee KA, Chandran V, Gladman DD (2010). Frequency, predictors, and prognosis of sustained minimal disease activity in an observational psoriatic arthritis cohort. Arthritis Care Res.

[CR4] Coates LC, Fransen J, Helliwell PS (2010). Defining minimal disease activity in psoriatic arthritis: a proposed objective target for treatment. Ann Rheum Dis.

[CR5] Cox DR, Snell EJ (1981). Applied statistics.

[CR6] Farewell VT, Su L (2011). A multi-state model for events defined by prolonged observation. Biostatistics.

[CR7] Harel O, Schafer JL (2009). Partial and latent ignorability in missing-data problems. Biometrika.

[CR8] Jackson CH (2011). Multi-state models for panel data: the msm package for R. J Stat Softw.

[CR9] Jackson CH, Su L, Gladman DD, Farewell VT (2016). On modelling minimal disease activity. Arthritis Care Res.

[CR10] Kalbfleisch JD, Lawless JF (1985). The analysis of panel data under a Markov assumption. J Am Stat Assoc.

[CR11] Lystig TC, Hughes JP (2002). Exact computation of the observed information matrix for hidden Markov models. J Comput Graph Stat.

[CR12] R Development Core Team (2010) R: a language and environment for statistical computing. R Foundation for Statistical Computing, Vienna, Austria. http://www.R-project.org, ISBN 3-900051-07-0. Accessed 18 Jan 2019

[CR13] Satten GA, Longini IM (1996). Markov chains with measurement error: estimating the true course of a marker of the progression of human immunodeficiency virus disease. Appl Stat.

[CR14] Sweeting MJ, Farewell VT, De Angelis D (2010). Multi-state Markov models for disease progression in the presence of informative examination times: an application to hepatatis C. Stat Med.

[CR15] Titman AC, Sharples LD (2010). Semi-Markov models with phase-type sojourn distributions. Biometrics.

[CR16] Tom BDM, Farewell VT (2011). Intermittent observation of time-dependent explanatory variables: a multi-state modelling approach. Stat Med.

[CR17] Wells GA, Boers M, Shea B, Brooks PM, Simon LS, Strand CV (2005). Minimal disease activity for rheumatoid arthritis: a preliminary definition. J Rheumatol.

